# Universal method to determine acidic licit and illicit drugs and personal care products in water by liquid chromatography quadrupole time-of-flight

**DOI:** 10.1016/j.mex.2016.04.004

**Published:** 2016-04-13

**Authors:** María Jesús Andrés-Costa, Eric Carmona, Yolanda Picó

**Affiliations:** Environmental and Food Safety Research Group (SAMA-UV), Desertification Research Centre CIDE (CSIC-UV-GV), Faculty of Pharmacy, University of Valencia, Av. Vicent Andrés Estellés s/n, Burjassot, 46100 Valencia, Spain

**Keywords:** Non-target screening by SPE and UHPLC quadrupole time-of-flight, High resolution mass spectrometry, UHPLC, emerging contaminants, water, identification, quantification

## Abstract

Pharmaceuticals, illicit drugs and personal care products are emerging contaminants widely distributed in water. Currently, a number of solid-phase extraction (SPE) procedures followed by liquid chromatography tandem mass spectrometry (LC–MS/MS) have been reported. However, target analysis of selected compounds is commonly used whereas other related contaminants present in the sample remain invisible. Carmona et al. [1] described a method for determining 21 emerging contaminants by LC–MS/MS with improved mobile phases. We tested this protocol in combination with high resolution mass spectrometry using a quadrupole time-of-flight (QqTOF) instrument to get a wide non-target screening approach in order to have a broader scope and more practical method for detecting licit and illicit drugs and personal care products than traditional target methods. The essential points in the method are:

• The screening capabilities of QqTOF (ABSciex Triple TOF™) are used for detecting and identifying non-target pharmaceuticals and a large number of other emerging contaminants in water.
• The quantitative features of the instrument, the Achilles heel of the QqTOF mass spectrometers, are established for few selected compounds.
• The method may be applied to identify a large number of emerging contaminants in water. However, pre-validation will be needed to quantify them.

## Graphical abstract

**Figure:**
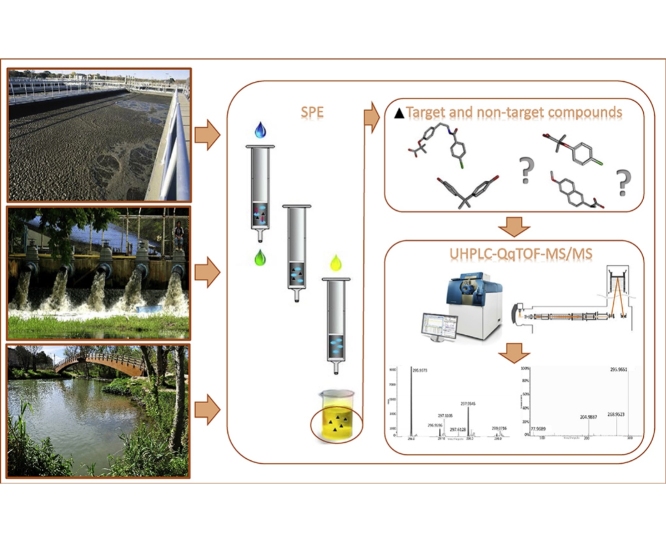

## Method details

Many different types of pollutants have been found in environmental compartments as water. Licit and illicit drugs or personal care products are some of the so-called emerging contaminants extensively used by humans [1], [2]. A number of analytical methods are already available [3], [4], [5], [6], [7], [8], [9] to determine emerging contaminants in environmental matrices at low concentrations. However, these methods are only reported for one type of instrument. In this study, we proposed a procedure to analyse pharmaceuticals, illicit drugs, personal care products and others contaminants on different water matrices through a common method for a triple quadrupole (QqQ) and a quadrupole time-of-flight (QqTOF) mass spectrometers.

### Reagents and materials

Acetaminophen, bezafibrate, bisphenol A, butylparaben, chloramphenicol, clofibric acid, diclofenac, ethylparaben, flufenamic acid, gemfibrozil, ibuprofen, indomethacin, methylparaben, naproxen, propylparaben, salicylic acid, thiamphenicol, triclocarban, triclosan and warfarin from Sigma-Aldrich (The Woodlands,Texas, USA) and tetrahydrocannabinol (THC) and 11-nor-9-carboxy-<DELTA>9-tetrahydrocannabinol (THC-COOH) from LoGiCal (Luckenwalde, Germany) were used as target analytes for QqQ analysis. Calibration standards were prepared by serial dilution of the mixed working solution. Stock and working solutions were stored at −20 °C in the dark [10].

Water used for preparation of calibration standards and LC–MS mobile phase was purified by an Elix Milli-Q system (Millipore, Billerica, MA, USA). Methanol was purchased from Panreac (Castellar del Vallès, Barcelona, Spain) and formic acid was purchased from Amresco (Solon, OH, USA). Ammonium fluoride was acquired from Alfa Aesar GmbH & Co KG (Karlsruhe, Germany).

### Extraction procedure

(1) Vacuum filter the samples (250 mL) through 0.45 μm retention capacity glass fiber filter of 90 mm diameter by Advantec (Toyo Roshi Kaisha, Ltd., Japan) using a Bücher funnel (with the filter) over a 250 mL Kitasato flask with 400 mbar h^−1^ Pa^−1^ of vacuum, to remove solid particles before the solid phase extraction (SPE).
(2) Put the Phenomenex Strata-X 33u Polymeric Reversed Phase (200 mg/6 mL) cartridges (Phenomenex, Torrance, Ca, USA) into a 12 port vacuum manifold Supelco Visiprep 57030-U of Sigma-Aldrich (St. Louis, MO, EEUU).
(3) Condition the cartridge with 6 mL methanol and 6 mL of Milli-Q water both with 400 mba h^−1^ Pa^−1^ vacuum.
(4) Pass the samples through the cartridges under previous vacuum at a flow rate of 10 mL min^−1^.
(5) Wash the cartridges with 6 mL of Milli-Q water.
(6) Dry the cartridges under vacuum for 15 min.
(7) Elute the analytes on a 15 mL Falcon tube VWR (Radnor, PA, USA) with 6 mL of methanol and then 3 mL of a methanol–dichloromethane solution (1:1, v/v) at gravity flow.
(8) Evaporate the extracts to dryness at 40 °C using a combined sample concentrator model SBHCONC/1 and a heating plate model SBH130D/3 both manufactured by Stuart^®^ (Stafford, UK).
(9) Redissolve the residue in 1 mL of water-methanol (70:30, v/v) by agitation and ultrasonication for 1 min and pass the extract to 2 mL amber vials with stoppers 99 mm + Septum Sil/PTFE, both manufactured by Análisis Vínicos S.L. (Tomelloso, Spain).

### UHPLC-QqTOF-MS/MS conditions

The chromatography was performed with an Agilent 1260 Infinity (Agilent, Waldbronn, Germany) using an Agilent Poroshell EC-C18 maintained at temperature of 30 °C. A constant flow rate of 0.2 mL min^−1^ was used. The mobile phase consists of two solvents, 2.5 mM ammonium fluoride in methanol (as organic solvent) and 2.5 mM ammonium fluoride in water (as aqueous solvent). The UHPLC system was coupled to a hybrid QqTOF ABSciex Triple TOF™ 5600 (Framingham, MA, USA). The MS acquisition was performed using negative ionization (NI) and scan mass spectra between *m*/*z* 100–700 with the Turbo Ionspray source. The MS parameters were: ion spray voltage, 5000 V; declustering potential (DP), 120 V; collision energy (CE), 10; temperature 400 °C with curtain gas (CUR) 25 (arbitrary units); ion source gas 1 (GS1) 50 and ion source gas 2 (GS2) 50. The QqTOF-MS/MS instrument was calibrated after every three samples using external reference compounds. The MS/MS acquisition was also performed using information-dependent acquisition (IDA) following operating parameters: declustering potential two (DP2), 110 V; ion release delay (IRD), 67 V; ion release width (IRW), 25 V; IDA MS/MS was performed at a fixed CE of 40 V, ions that exceeded 100 cps and ion tolerance of 50 mDa (isotopes higher than 4 Da were excluded). Data acquisition and processing was carried out using software Analyst (Framingham, MA, USA), Peak View 1.0 with the application XIC manager and MultiQuant 2.0.

### Sampling

The developed method was applied to 21 influent and 21 effluent samples collected from three wastewater treatment plants (WWTPs) of metropolitan area of Valencia and 25 surface waters from Túria River. Wastewater samples were 24-h composite samples and river samples were grab ones. All samples were stored in polyethylene terephthalate (PET) bottles and once arrived at the laboratory, immediately frozen at −20 °C until analysis to prevent degradation of contaminants.

### Validation of the analytical method

Validation of the analytical method was performed partly according to the Commission Decision 2002/657/EC [11] and partly to the Eurachem guide [12] on that subject since none of them has a binding nature for water contaminants.

Table 1 shows limit of quantification (LOQ), matrix effect (ME), recovery and relative standard deviation (RSD) obtained by UHPLC-QqTOF determination. The method provides LOQ between 1 and 150 ng L^−1^, recoveries from 39% to 115%, matrix effects ranged from 6 to −52% and relative standard deviations (RSD) lower than 21%. The linearity was determined by calibration curves from LOQ- 5000 ng L^−1^ in water-methanol (70:30) or as a matrix matched standards, with linear coefficients of determination (R^2^) ≥ 0.99, except for salicylic acid (R^2^) ≥ 0.98. Table **S1** in Supplementary information depicts these parameters for UHPLC-QqQ.

Table 2 shows the quantification of the selected analytes in the different water samples, as mean value ± RSD using QqQ and QqTOF instruments. The quantification of the detected compounds in the three matrices with QqQ was carried out according to the instrumental conditions previously reported [1] (see Table **S2** in Supplementary information). The quantification of detected compounds with QqTOF was performed using MultiQuant 2.0 software. The results of QqQ and QqTOF were very similar, which confirms that the method is valid for both.

**Fig. 1:**
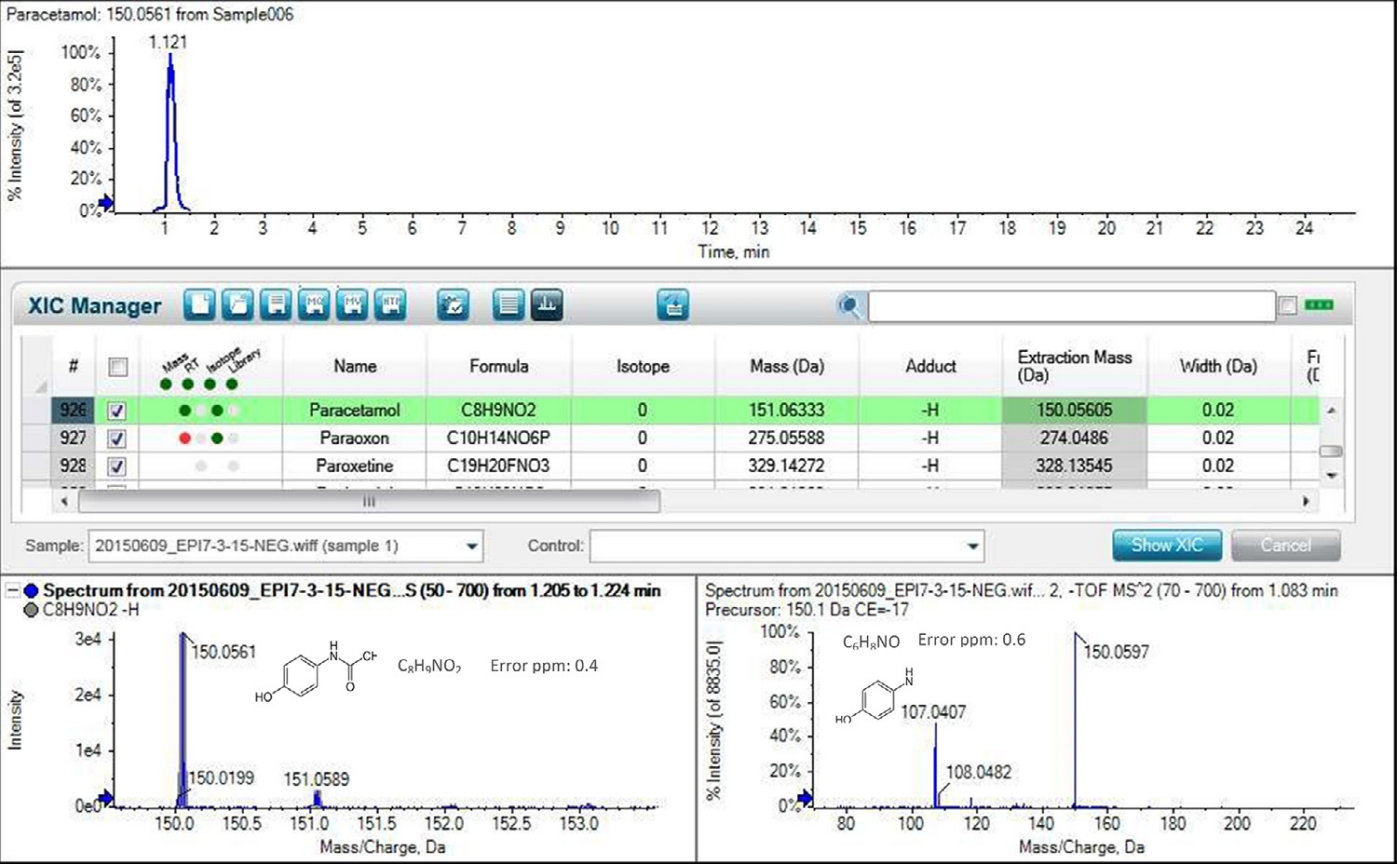
MS and MS/MS Spectra of target analyte acetaminophen (paracetamol).

**Fig. 2:**
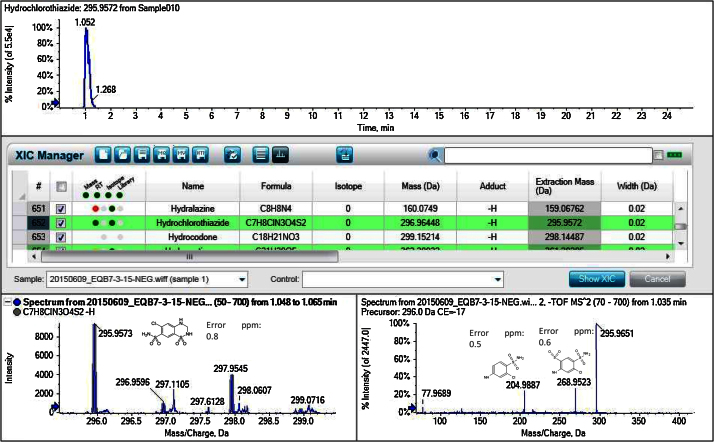
MS and MS/MS Spectra of non-target analyte hydrochlorothiazide.

Table 3 presents, mass (Da), adduct, extraction mass (Da), mass error (ppm), retention time (RT) and intensity of the selected compounds (spiked Milli-Q water with 100 ng L^−1^). The identification of target and non-target was carried out against the XIC manager Table with data of 1212 pharmaceuticals, 546 pesticides, 378 polyphenols and 233 mycotoxins. Furthermore, a total of 86 ± 9 pharmaceuticals, 2 ± 1 pesticides and 14 ± 3 other compounds were detected in influent samples; 45 ± 14 pharmaceuticals, 1 ± 1 pesticides and 7 ± 3 other compounds were detected in effluent samples, and 20 ± 6 pharmaceuticals, 1 ± 1 pesticides and 5 ± 3 other compounds in river water samples. Fig. 1 illustrates the identification of acetaminophen (paracetamol) and Fig. 2 of the non-selected hydrochlorothiazide to show the identification system capabilities. Fig. **S1** in Supplementary information shows the extracted ion chromatogram of all substances present in water and the non-target compound identification of theophylline in influent wastewater sample.

## Additional information

### Background

There are hundreds, even thousands of emerging contaminants that can occur in water. Traditionally, the scheme used for their determination involves generic sample preparation procedures able to extract almost any of them, and target determination for the unique and highly specific detection of the selected contaminant(s) [3], [4], [5]. This scheme is time-consuming (ca. 30 min each chromatographic run for a specific group of contaminants) and do not have versatility to detect unexpected emerging contaminants not selected for the target analysis. Currently, there are some reports of non-target detection through high resolution mass spectrometry that provide full scan information as well as compound fragmentation (any *m*/*z* signal from the sample extract) [2], [8]. However, high resolution mass spectrometer can provide inaccurate quantification [8] or enough sensitivity [2]. Latest generation instruments have improved their quantification possibilities as well as the identification capabilities of any unexpected substance by the application information dependent acquisition (IDA) modes that automatically provide MS/MS spectra of the most intense precursor ions (without previous selection) as an additional confirmation of the detected compounds [2].

The few examples of these broad screening systems are mostly focus on the positive ionization mode because there are more contaminants that ionized in positive mode and their MS sensitivity is higher. When mass spectrometry is combined with liquid chromatography (recommended for polar compounds as the emerging contaminants) the commonly used additives of the mobile phases (volatile salts and acids) enhanced the ionization in the positive ionization mode and inhibited it in the negative ionization one. Acidic contaminants, commonly better ionized by negative ionization are more difficult to detect and frequently the sensitivity does not reach the low levels emerging contaminants are present in water. Recently, Petrie et al. [9] demonstrated a substantial improvement of ionization efficiency in negative ionization mode by using NH_4_F enriched mobile phase to metabolomics studies. Our previously reported method using NH_4_F as mobile phase additive instead of more conventional substances also improved the ionization efficiency of the 21 selected compounds in a reproducible way using a triple quad instrument [1]. These results were recently confirmed for wide range of compounds [10]. Our current study proves that the addition of NH_4_F to the mobile phase instead of more conventional ammonium formate is also successful for the simultaneous determination of acidic contaminants in water by UHPLC-QqTOF [13], [14] increasing sensitivity and quantification capabilities. The strong basicity of the fluoride anion (F^−^) in the gas phase increases deprotonation of basic analytes.

The results showed good agreement between both systems for the analysed samples. For QqQ, naproxen was the pharmaceutical at highest concentration (3327 ng L^−1^) at the influent of the WWTPs which was in a lower concentration at the effluent (10 ng L^−1^). Indomethacin, clofibric acid and triclocarban were the lowest detected with 7 ng L^−1^ in influent samples. Regarding effluent samples, the highest detected concentration was diclofenac with 173 ng L^−1^, being the gemfibrozil the compound with the lowest (5 ng L^−1^). Finally, for river waters, the concentration of target analytes was, in general, lower than WWTPs samples being the compound in major concentration the acetaminophen with 177 ng L^−1^ and ibuprofen with 153 ng L^−1^. Concerning the concentration calculated with QqTOF, the mean concentration levels detected in influent samples ranged from 12 ng L^−1^ (clofibric acid) to 2963 ng L^−1^ (naproxen) being naproxen the most detected compound as in the case of QqQ. In the effluent the highest concentrations were methylparaben (121 ng L^−1^) followed by diclofenac (109 ng L^−1^). In river waters the concentration levels ranged from 7 ng L^−1^ (butylparaben) to 159 ng L^−1^ (ibuprofen). These results show a good correlation between both techniques as in our previous paper [3].

## Figures and Tables

**Table 1:** Method performance parameters: limit of quantification (LOQ, ng L^−1^), absolute recoveries (%), method repeatability (RSD, %) and matrix effect (ME, %) using QqTOF for effluent, influent and river water samples.

Analyte	WWTP Influent				WWTP Effluent				River water			
	LOQ (ng L^−1^)	Recovery (%)	RSD (%)	ME (%)	LOQ (ng L^−1^)	Recovery (%)	RSD (%)	ME (%)	LOQ (ng L^−1^)	Recovery (%)	RSD (%)	ME (%)
Acetaminophen	30	84	15	−33	15	86	14	−26	15	95	10	−12
Bezafibrate	30	75	15	−32	20	78	13	−28	10	85	11	−16
Bisphenol A	20	80	19	−12	10	80	12	−10	5	89	17	−18
Butylparaben	10	79	17	−19	5	101	18	−19	5	115	12	−10
Chloramphenicol	50	62	11	−36	20	75	17	−32	20	92	10	−23
Clofibric acid	100	61	12	−41	30	70	21	−31	20	76	20	−31
Diclofenac	150	82	10	−47	40	91	15	−45	30	98	12	−15
Ethylparaben	50	81	13	−31	25	95	11	−35	20	96	18	−28
Flufenamic acid	40	71	14	−29	30	69	15	−18	5	89	15	−16
Gemfibrozil	10	61	9	−29	10	67	12	−20	10	78	17	−9
Ibuprofen	100	80	11	−32	80	92	18	−15	50	90	12	−11
Indomethacin	50	78	15	−15	50	98	10	−11	30	79	13	−2
Methylparaben	30	80	9	−33	10	90	12	−35	5	89	20	−19
Naproxen	50	71	17	−30	20	85	18	−32	30	89	17	−21
Propylparaben	50	71	21	−31	5	81	13	−24	10	102	13	−5
Salicylic Acid	100	39	10	−52	50	62	18	−39	20	61	25	−13
THC	50	48	18	−9	20	52	17	−10	10	54	19	−6
THC-COOH	10	50	9	−19	10	63	14	−19	5	62	15	6
Thiamphenicol	120	74	11	−21	100	92	19	−20	80	89	18	−7
Triclocarban	50	85	13	−19	5	79	15	−21	5	91	14	5
Triclosan	20	82	19	−10	20	91	15	2	10	76	15	−12
Warfarin	30	73	8	−11	20	84	12	−22	1	86	13	−13

Linearity: linear coefficients (R^2^) were ≥ 0.99 in all cases, except for salicylic acid (R^2^ ≥ 0.98); LOQ was established as the concentration that, after extraction, gives a UHPLC peak height value 1.0 × 10^4^; Recoveries and relative standard deviations (RSDs) of selected compounds were calculated in samples spiked at 100 ng L^−1^ subtracting the peak areas corresponding to native analytes in the sample and tested in quintuplicate; Matrix effect was evaluated by comparing the slope of the calibration curves obtained for spiked influent, effluent or surface water extracts with the slope of that obtained for standard prepared in water-methanol (70:30, v/v) spiked at the same level.

**Table 2:** Comparison of the quantitative results obtained using the ABSciex TripleTOF™ 5600 (QqTOF) and a more traditional triple quadrupole (QqQ) for influent, effluent and river water samples.

Compounds^a^	Sample 1 Influent (ng L^−1^)^b^		Sample 2 Effluent (ng L^−1^)^b^		Sample 3 River Water (ng L^−1^)^b^	
	QqTOF	QQQ	QqTOF	QQQ	QqTOF	QQQ
Acetaminophen	2114	2497	31	21	139	177
Bezafibrate	35	47	11	15	12	7
Bisphenol A	495	571	96	72	36	41
Butylparaben	35	22	n.d.	n.d.	7	5
Chloroamphenicol	n.d.	n.d.	n.d.	n.d.	62	68
Clofibric acid	12	7	n.d.	n.d.	n.d.	n.d.
Diclofenac	296	331	109	173	39	33
Ethylparaben	99	113	49	71	n.d.	6
Flufenamic acid	75	90	39	48	29	22
Gemfibrozil	105	155	n.d.	5	31	34
Ibuprofen	1796	1978	n.d.	n.d.	159	153
Indomethacin	n.d.	7	n.d.	18	n.d.	n.d.
Methylparaben	259	331	121	99	19	24
Naproxen	2963	3327	21	10	38	36
Propylparaben	494	519	36	45	11	12
Salicylic acid	596	778	n.d.	n.d.	29	22
THC	n.d.	n.d	n.d.	n.d.	n.d.	n.d.
THC-COOH	409	592	n.d.	n.d.	21	23
Thiamphenicol	n.d.	n.d.	n.d.	n.d.	n.d.	10
Triclocarban	n.d.	7	n.d.	n.d.	n.d.	n.d.
Triclosan	752	926	n.d	n.d.	n.d.	n.d.
Warfarin	n.d.	11	29	31	33	54

n.d.: non-detected.

a Only analytes that occur in any of the samples.

b Average (SD) n = 3.

**Table 3:** Experimental parameters used for the identification of the target analytes (n = 5).

Name	Mass (Da)	Adduct	Extraction Mass (Da)	Found at mass (Da)	Error ppm	Error (mDa)	Found at RT (min)	Intensity
Acetaminophen	151.06333	−H	150.05605	150.05612	0.4	0.3	1.12	35326
Bezafibrate	361.10809	−H	360.10427	360.10409	−0.8	−0.2	14.36	40634
Bisphenol A	228.11504	−H	227.11496	227.11431	−2	−0.7	14.86	73687
Butylparaben	194.09430	−H	193.09421	193.09438	0.8	0.2	13.31	70035
Chloramphenicol	322.01233	−H	321.01129	321.01174	1.2	0.4	10.38	63257
Clofibric acid	214.03967	−H	213.03037	213.02899	−4.2	−1.4	9.89	55963
Diclofenac	295.01669	−H	294.01596	294.01617	0.6	0.2	15.87	75981
Ethylparaben	166.06299	−H	165.06196	165.06323	3.3	1.3	12.36	62257
Flufenamic acid	281.06636	−H	280.05909	280.05942	1.2	0.3	14.63	45704
Gemfibrozil	250.15689	−H	249.14962	249.1498	0.7	0.2	14.59	64434
Ibuprofen	206.13068	−H	205.1234	205.12357	0.8	0.2	14.52	70035
Indomethacin	357.07678	−H	356.07536	356.07640	2.9	2.9	16.25	59363
Methylparaben	152.04735	−H	151.04631	151.04657	0.9	0.9	9.64	61259
Naproxen	230.09430	−H	229.09411	229.09489	2.6	2.6	13.91	79632
Propylparaben	180.07864	−H	179.07796	179.07803	0.4	0.4	14.47	42963
Salicylic acid	138.03169	−H	137.03165	137.03172	0.4	0.4	2.56	49332
THC	314.22458	−H	313.2173	313.21728	−0.1	0	16.11	44379
THC-COOH	344.19876	−H	343.19148	343.19193	1.3	0.4	14.63	73637
Thiamphenicol	355.00479	−H	354.00432	354.00499	1.5	1.5	2.67	75336
Triclocarban	313.97806	−H	312.97124	312.97111	−0.4	−0.4	15.63	48525
Triclosan	287.95117	−H	286.90985	286.91012	1	1	16.57	71225
Warfarin	308.10486	−H	307.10362	307.10348	−0.4	−0.4	10.78	79325

RT: retention time.
