# Supplementary material for: Universal method to determine acidic licit and illicit drugs and personal care products in water by liquid chromatography quadrupole time-of-flight
Source: MethodsX. 2016 Apr 13;3:307–14. doi: 10.1016/j.mex.2016.04.004 (PMC4840423; doi:10.1016/j.mex.2016.04.004)
Supplement: Supplementary file 1 [file mmc1.docx]

**Supplementary information**

**Title: “Universal method to determine acidic licit and illicit drugs and personal care products in water by liquid chromatography Quadrupole Time-of-Flight”**

María Jesús Andrés-Costa^1^, Eric Carmona^1^ and Yolanda Picó^1^

^1^Environmental and Food Safety Research Group (SAMA-UV), Desertification Research Centre CIDE (CSIC-UV-GV), Faculty of Pharmacy, University of Valencia, Av. Vicent Andrés Estellés s/n, Burjassot, 46100 Valencia, Spain

*Corresponding authors. Tel.: + 34 963543092; fax: + 34 963544954

E-mail: [M.Jesus.Andres@uv.es](mailto:M.Jesus.Andres@uv.es)

Website: sama-uv.es

**Table S1**. UHPLC-QqQ-MS/MS conditions for target compounds.

| Compound | Molecular formula | RT (min) | SRM1 | Frag/CE(V) | SRM2 | Frag/CE(V) |
| --- | --- | --- | --- | --- | --- | --- |
| Acetaminophen | C_8_H_9_NO_2_ | 12,1 | 150 > 108 | 88/14 | -- | -- |
| Bezafibrate | C_19_H_20_ClNO_4_ | 13,3 | 360 > 274 | 106/10 | 360 > 154 | 106/22 |
| Bisphenol A | C_15_H_16_O_2_ | 13,8 | 227 > 212 | 138/14 | 227 > 133 | 138/25 |
| Butylparaben | C_11_H_14_O_3_ | 14,6 | 193 > 137 | 122/10 | 193 > 92 | 122/10 |
| Chloramphenicol | C_11_H_12_Cl_2_N_2_O_5_ | 8,4 | 321 > 152 | 128/10 | 321 > 176 | 128/10 |
| Clofibric acid | C_10_H_11_ClO_3_ | 9.2 | 213 > 127 | 76/1 | 213 > 35 | 76/33 |
| Diclofenac | C_14_H_11_Cl_2_NO_2_ | 14,5 | 294 > 250 | 88/10 | 294 > 178 | 88/22 |
| Ethylparaben | C_9_H_10_O_3_ | 11,8 | 165 > 92 | 103/10 | 165 > 137 | 103/22 |
| Flufenamic Acid | C_14_H_10_F_3_NO_2_ | 15,1 | 280 > 236 | 106/10 | 280 > 176 | 106/30 |
| Gemfibrozil | C_15_H_22_O_3_ | 16,5 | 249 > 121 | 88/10 | 249 > 127 | 88/20 |
| Ibuprofen | C_13_H_18_O_2_ | 14,8 | 205 > 161 | 68/2 | 205 > 159 | 68/12 |
| Indomethacin | C_19_H_16_ClNO_4_ | 15,0 | 356 > 296 | 98/10 | 356 > 282 | 98/22 |
| Methylparaben | C_8_H_8_O_3_ | 9,1 | 151 > 92 | 93/10 | 151 > 136 | 93/18 |
| Naproxen | C_14_H_14_O_3_ | 12,0 | 229 > 170 | 88/10 | 229. > 169 | 88/26 |
| Propylparaben | C_10_H_12_O_3_ | 13,4 | 179 > 92 | 112/10 | 179 > 137 | 112/22 |
| Salicylic Acid | C_7_H_6_O_3_ | 2,1 | 137 > 93 | 86/10 | 137 > 64 | 86 / 30 |
| THC | C_21_H_30_O_2_ | 17,5 | 313 >191 | 186/26 | 313 >245 | 186/26 |
| THC COOH | C_21_H_28_O_4_ | 13,6 | 343>245 | 166/18 | 343 >299 | 166/20 |
| Thiamphenicol | C_12_H_15_Cl_2_NO_5_S | 2,3 | 354 > 290 | 128/10 | 354 > 64 | 128/74 |
| Triclocarban | C_13_H_9_Cl_3_N_2_O | 16,8 | 313 > 160 | 86/10 | 313 > 126 | 86/10 |
| Triclosan | C_12_H_7_Cl_3_O_2_ | 16,9 | 287 > 35 | 98/14 | 289 > 35 | 98/13 |
| Warfarin | C_19_H_16_O_4_ | 11,8 | 307 > 161 | 136/10 | 307 > 117 | 136/30 |
| RT: Retention Time; SRM: Selected Reaction Monitoring; Frag: Fragmentor; CE: Collision Energy | | | | | | |

**Table S2**. Method performance parameters: limit of quantification (LOQ, ng L^-1^), absolute recoveries (%), method repeatability (RSD, %) and matrix effect (ME, %) of QqQ for effluent, influent and River water samples.

| Analyte |  | WWTP Influent | | | |  | WWTP Effluent | | | |  | River water | | | |
| --- | --- | --- | --- | --- | --- | --- | --- | --- | --- | --- | --- | --- | --- | --- | --- |
|  |  | LOQ  (ng L^-1^) | Recovery  (%) | RSD  (%) | ME  (%) |  | LOQ  (ng L^-1^) | Recovery  (%) | RSD  (%) | ME  (%) |  | LOQ  (ng L^-1^) | Recovery  (%) | RSD  (%) | ME  (%) |
| Bezafibrate |  | 5 | 71 | 18 | -25 |  | 1 | 76 | 16 | -18 |  | 1 | 80 | 11 | -10 |
| Bisphenol A |  | 2 | 80 | 15 | -18 |  | 1 | 83 | 10 | -20 |  | 1 | 82 | 17 | -10 |
| Butylparaben |  | 0,7 | 86 | 15 | -25 |  | 0.5 | 98 | 15 | -19 |  | 0.8 | 105 | 12 | -7 |
| Chloramphenicol |  | 15 | 62 | 12 | -40 |  | 6.5 | 69 | 15 | -26 |  | 5 | 80 | 10 | -25 |
| Chlorfibric acid |  | 10 | 71 | 13 | -45 |  | 3 | 79 | 20 | -30 |  | 2 | 77 | 20 | -20 |
| Diclofenac |  | 18 | 80 | 16 | -45 |  | 4 | 88 | 11 | -40 |  | 4 | 100 | 12 | -30 |
| Ethylparaben |  | 5 | 89 | 21 | -35 |  | 1.5 | 99 | 18 | -20 |  | 1 | 98 | 18 | -15 |
| Flufenamic Acid |  | 4 | 70 | 15 | -20 |  | 0,6 | 70 | 13 | -7 |  | 0.5 | 83 | 15 | -5 |
| Gemfibrozil |  | 10 | 65 | 14 | -25 |  | 1.5 | 72 | 11 | -7 |  | 1 | 77 | 17 | 0 |
| Ibuprofen |  | 26 | 87 | 22 | -20 |  | 20 | 96 | 17 | -10 |  | 15 | 93 | 12 | -4 |
| Indomethacin |  | 15 | 68 | 14 | -17 |  | 5 | 72 | 20 | -8 |  | 5 | 75 | 13 | -5 |
| Methylparaben |  | 6 | 86 | 12 | -50 |  | 1 | 91 | 18 | -35 |  | 1 | 91 | 20 | -15 |
| Naproxen |  | 6 | 77 | 20 | -35 |  | 2 | 86 | 13 | -20 |  | 3 | 86 | 17 | -12 |
| Propylparaben |  | 5 | 91 | 25 | -30 |  | 0.5 | 94 | 12 | -25 |  | 0,5 | 111 | 13 | -10 |
| Salicylic acid |  | 15 | 38 | 13 | -65 |  | 1 | 49 | 19 | -40 |  | 0.5 | 49 | 25 | -20 |
| THC |  | 2 | 47 | 20 | -17 |  | 0.5 | 50 | 17 | -15 |  | 0.4 | 55 | 19 | 2 |
| THC COOH |  | 1 | 55 | 15 | -20 |  | 1 | 60 | 15 | -15 |  | 0,6 | 59 | 15 | 0 |
| Thiamphenicol |  | 12 | 79 | 19 | -40 |  | 14 | 82 | 20 | -25 |  | 15 | 87 | 18 | -15 |
| Triclocarban |  | 5 | 83 | 17 | -15 |  | 0.5 | 89 | 14 | -10 |  | 0.3 | 99 | 14 | 0 |
| Triclosan |  | 2 | 91 | 18 | -13 |  | 5 | 95 | 15 | -12 |  | 4 | 100 | 15 | 0 |
| Warfarin |  | 5 | 79 | 10 | -15 |  | 1 | 83 | 13 | -20 |  | 0.7 | 81 | 13 | -5 |

**Figure S1.** **a)** Extracted ion chromatogram of the non-target compounds, **b)** theophylline in influent wastewater sample against the XIC manager Table with data on 1212 pharmaceuticals, 546 pesticides, 378 polyphenols and 233 mycotoxins.

**a)**


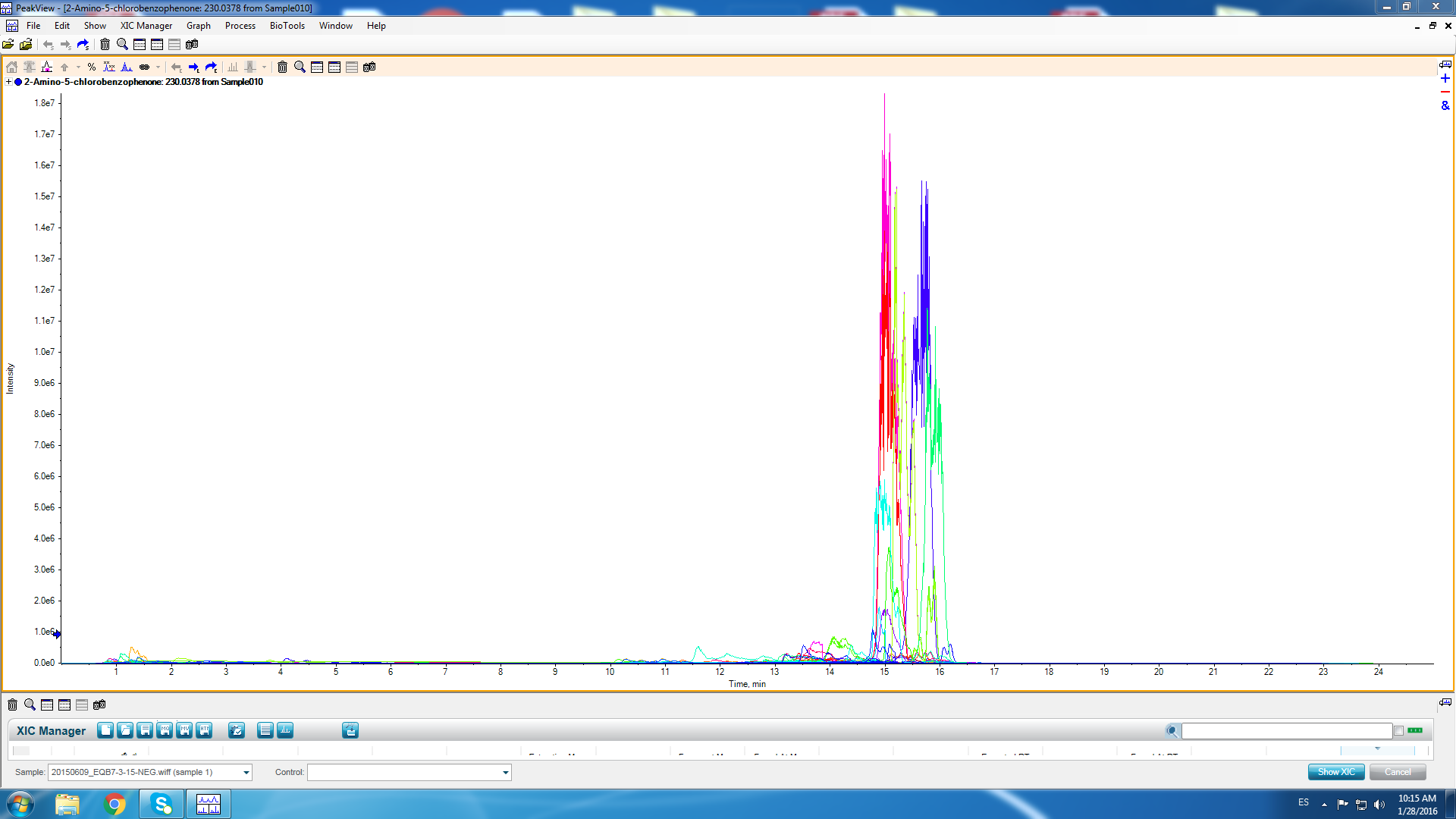

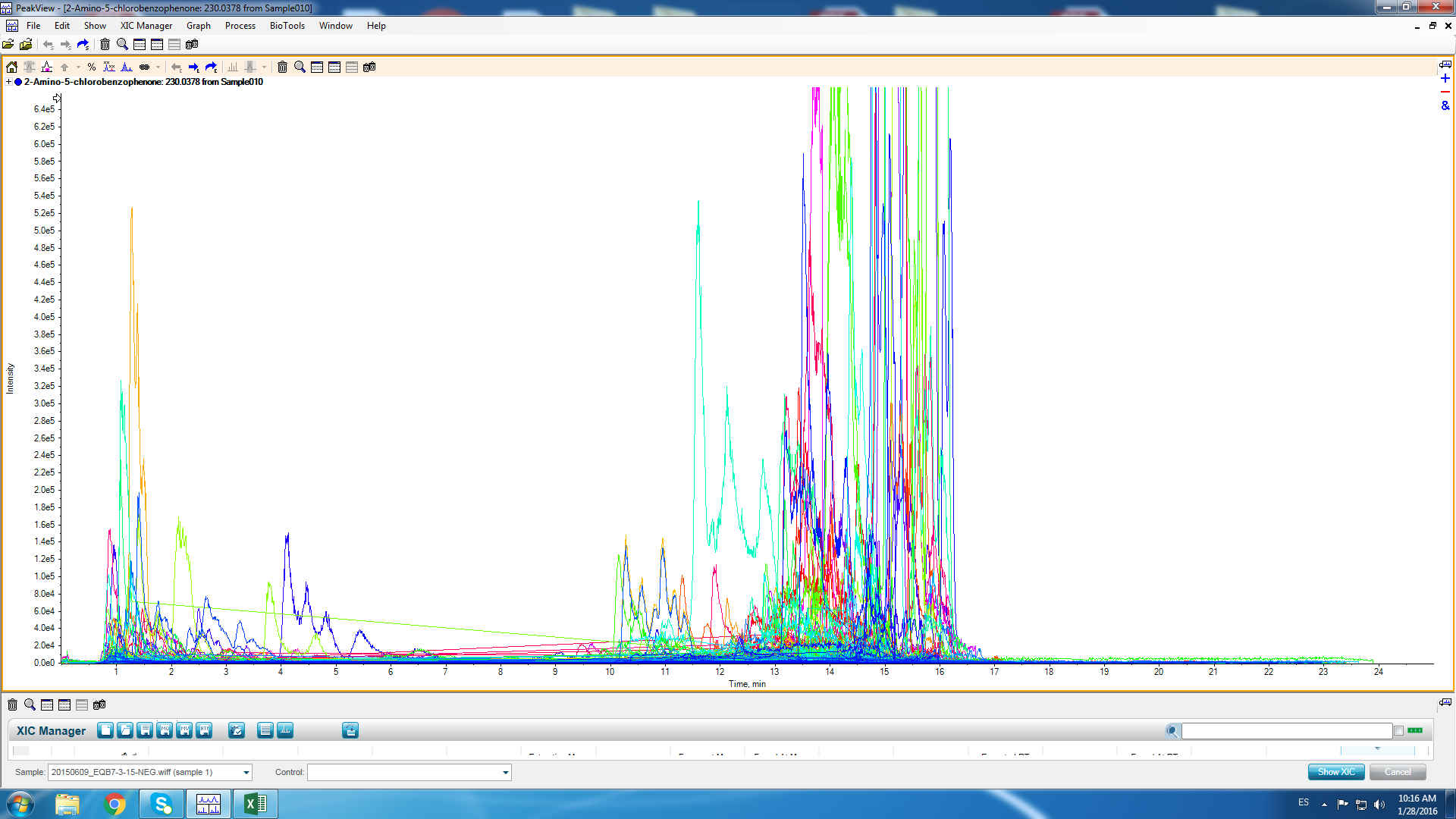


**b)**

C_7_H_8_N_4_O_2_

Error ppm: 0.8


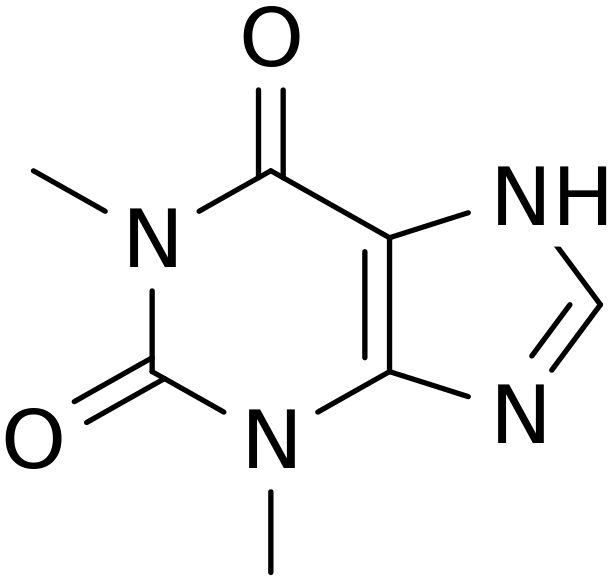

Error ppm: 0.3

C_8_H_9_NO_2_

Error ppm: 0.4


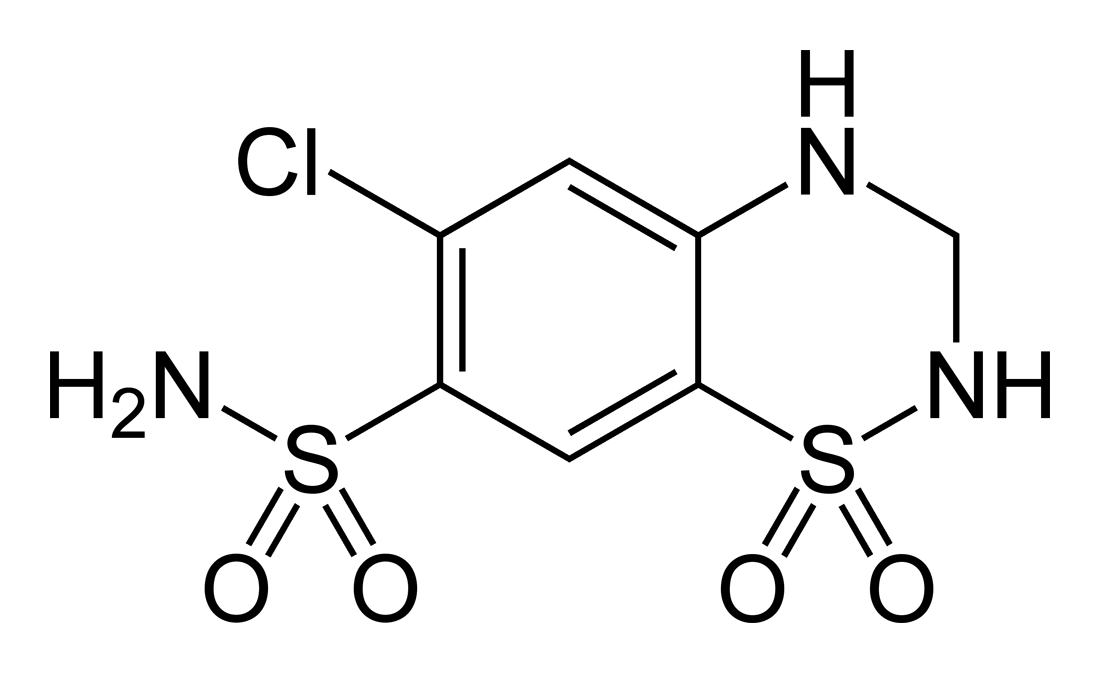


C_5_H_4_N_3_O
